# Public perceptions uncovered: engaging in decision-making regarding non-pharmaceutical interventions: Sophie Kemper

**DOI:** 10.1093/eurpub/ckac130.220

**Published:** 2022-10-25

**Authors:** S Kemper, MEJ Bongers, JFH Kupper, M De Vries, A Timen

**Affiliations:** 1 Centre for Communicable Disease Control, RIVM, Bilthoven, Netherlands; 2 Athena Institute, VU University Amsterdam, Amsterdam, Netherlands

## Abstract

**Background:**

To control the COVID-19 pandemic, non-pharmaceutical interventions (NPIs) were implemented worldwide, that heavily impacted the daily lives of citizens. Occasionally, the public expressed discontent about NPIs, as NPIs did not always corresponded with their preferences. The question is if and how public engagement (PE) could aid in development and implementation of NPIs, in order to improve legitimacy, quality and compliance.

**Methods:**

An online survey was conducted from 27 October to 9 November 2021, with a representative sample of the public in the Netherlands on gender, age, education, place of residency and migration background. In total 4981 respondents participated. Perceptions and preferences about PE in decision-making on NPIs to control COVID-19 was collected. Four NPIs were used: Nightly curfew (NC); Digital Covid Certificate (DCC); Closure of schools and daycares (CSD); and 1.5meter social distance.

**Results:**

Around 25% of respondents expressed a desire to engage in decision-making, as it would increase understanding and quality of NPIs, and their trust in the government. Especially for the NPIs DCC and NC, respondents found it valuable to engage, by providing their perspective on certain trade-offs in values in decision-making (e.g. opening up society vs division in society by vaccination status). The public could play a role by giving feedback on bottlenecks during decision-making, however overall responsibility should stay with experts and policy-makers. Desire for engagement was lowest for CSD. Around 50% of the respondents did not want to engage, as they felt they were not knowledgeable enough to do so and did not perceive a need to engage. The other 25% had a neutral disposition.

**Conclusions:**

Engagement was not self-evident for most respondents, yet the ones willing to engage revealed important possibilities for future outbreaks. Next, a deliberative process for PE in decision-making could be executed, in order to implement our findings in practice.

**Key messages:**

- Although engagement was not self-evident, respondents who were willing to engage revealed important possibilities to shape future public engagement practices in decision-making in COVID-19 control.
- Respondents were mostly willing to engage in NPIs with no consensus in the trade-offs of relevant values in decision-making, such as the Nightly Curfew and the Digital Covid Certificate.

